# Editorial Notes

**Published:** 1908-12

**Authors:** 


### Editorial Notes.

**University College, Bristol, Faculty of Medicine.**

THE annual distribution of prizes took place on October 1st, again under the chairmanship of the Right Hon. Lewis Fry, the distribution being made by Sir Rubert Boyce, F.R.S., Holt Professor of Pathology in the University of Liverpool, who gave an address on the subject of University extension. He pointed out the leading part which the medical schools of this country had taken in the modern Universities, and he remarked that "it should be borne in mind that there was no city of the Empire which showed a longer or a worthier list of men of culture than Bristol. It was not to be wondered at that medical schools, no matter how modest in size, should have early seized the opportunity, when it came, of extending their sphere and reaching out a helping hand to colleagues organising and desirous of founding schools in other departments of thought. By this coalition of teaching forces there grew up in this country those institutions well known as University Colleges, which paved the way for the new Universities. This union proved of the greatest value to both sides; the views of both were broadened and stimulated, and this soon made itself felt throughout the country by the growing desire of the teachers in these colleges to attract the best men to their faculties and to beg money to endow and build laboratories for the better accommodation of students and teachers. And let it be said to their lasting credit and honour that nearly all of these colleges saw clearly that the most successful way to attain their object was to convert their colleges into Universities, the logical and natural conclusion to their long period of evolution. Thus it was that in the last ten years they had been privileged to witness a remarkable recrudescence of University learning in this country. Indeed, the twentieth century would be famous for the foundation of Universities in England, Ireland, Wales, and the Colonies, the like of which had no parallel in any other country, and which surely showed that public feeling had been thoroughly roused, and that the importance of University training for the public good was appreciated to the full by all classes of society. He proceeded to mention a few directions in which they might radiate out and take root with much profit to their faculty, and with great good to the public. A cancer investigation department, schools of research for the scientific co-ordinated study of epilepsy and lunacy, the same for the study of the simple infectious diseases, like scarlet fever, measles, &c. ; a special school of research for the study and advancement of their knowledge of tropical diseases, for which they in Bristol were eminently well placed, and towards which they had many of the necessary qualifications. Hygiene offered them many more fields. Let them also establish a school of economic entomology. The growth of this new science was marvellous. It embraced all those pests which bit and infected man and animals—flies, fleas, ticks, bugs, and parasites generally. It was indeed a practical study. Then, with the establishment of these divisions, together with their professors of botany, zoology, physiology, and chemistry, they would find that the next great natural and logical step would be to create a great school of veterinary medicine in the South-West of England, as well as a great agricultural school, these two working hand in hand. No one could say these were not sound, practical, and legitimate aspirations. There were many more—let them not forget their friends, the pharmacists ; organise them and create a good school. Gentlemen of the Bristol School of Medicine had a sumptuous harvest in front of them—let them muster their labourers and reap it.

**International Congress on Tuberculosis, Washington, 1908.**

The war against tuberculosis will have derived great strength from this Congress. Nothing before has been more popular and more universally accepted by all the peoples of the earth—physicians, laymen, of all varieties, philanthropists, and legislators of America, Europe, Africa, India, China and Japan, all united in a common cause to suppress a disease which has threatened to exterminate the human race. It is well known how great the mortality has been, but Professor Irving Fisher's estimates were a revelation to the world when he stated that consumption kills 138,000 in America yearly, and costs the nation two hundred million pounds, and further that the long drawn-out illness of most cases may be estimated to cost some eighty-eight millions a year besides. It is not so commonly known that the history of the coloured races in America shows that at the present rate certain tribes of negroes, with Sioux and other Indians, will soon be extinct. So also is it with the Irish race in America, which are almost as liable to consumption as the negro.

In the State of New York it is estimated that the loss, not only by deaths but by protracted sickness, amounts to some fifteen millions of dollars, as consumption accounts for every third death during the working period of life, and every other workman who becomes incapacitated, the loss by sickness being four times greater than by death.

The seriousness of this combat is shown by the awakening of general interest in the subject. The physicians of America have shown an enthusiasm which fully rivals that of their foreign guests at this Congress, and the resolutions adopted at this great meeting may be taken seriously, and not as recommendations due to the preaching of excited faddists.

The reduction of mortality which is now manifest may be claimed to be the result of modern hygienic methods, *e.g.* in Germany Professor Koch claims that the mortality has been reduced one-half during the last three decades, giving a gain of 30,000 lives to the State.

The veteran Dr. Trudeau rejoiced that the times have changed. He stated that for thirty-five years he had struggled against darkness and indifference, but that the true light shineth now. It is admitted that the life, habits and environment of the individual are all essential, that mere climate counts for little in the contest, that without hygiene climate is as sounding brass or a tinkling cymbal (Solly). It is also generally admitted that although it is impossible as yet to extirpate all tubercle bacilli, yet it is possible to so strengthen and harden the body that the absorbed germs cannot take hold upon it. Recovery, however, is at best difficult and too often doubtful. Prevention is a more thankful task.

**Report of British Committee, Washington.**

THE official report of the Executive Committee for Great Britain and Ireland—giving an account of certain of the more important steps taken in this country with the object of accelerating the decline in the death-rate from pulmonary tuberculosis, which has been in progress during the last fifty years—contains much material for study and reflection.

It has been drawn up and signed by Dr. C. Theodore Williams, Dr. H. Timbrell Bulstrode, and Dr. I. Walker Hall, who give an authentic and complete account of what is being done in this country towards the specific control of tuberculosis.

It is pointed out that the system of voluntary notification has not been altogether a success, and that even where compulsory notification has been tried it has not materially aided us in obtaining really early cases of the disease. There seems to be no question that, in so far as the patients are concerned, the detection of early cases is the greatest use to which notification can be applied. It seems to be too early to expect that any direct measures which have been taken as a consequence of notification should have already resulted in any appreciably added decline in the death-rate of the places in which such measures have been taken.

As regards the provision of sanatoria by public health authorities, the city of Bristol has taken quite a leading part in securing twenty beds at the Winsley Sanatorium. Contributions in varying degrees are made to this sanatorium by the sanitary authorities of Bath, Taunton, Swindon, and other towns, as also by certain rural district councils.

It is of interest to note that more recently the city of Birmingham has been the first municipal authority in this country to provide for its consumptive sick a separate institution, on Leckhampton Hill, near Cheltenham. It is suggested that a statue to Bodington might well be erected there in recognition of his inauguration near to Birmingham, some sixty years ago, of the first sanatorium in the world.

It has been universally found that the difficulties of securing early cases by existing methods are very great, more especially among the working classes, and schemes have been suggested for the searching out of early cases in the homes of the people through the agency of medical officers of health, lady almoners, district visitors, &c.; and when no hospital for the selection of cases exists, the best results are likely to accrue when the medical superintendent of the sanatorium, by visiting certain centres in his district at definite dates, is himself made responsible for such cases as he admits. Where medical referees exist, it would be understood that the function of such referees extends only to the recommendation for examination by the medical superintendent at the local centres. This would be the most satisfactory safe-guard if it were possible; but it would necessitate the frequent absence of the medical superintendent from his sanatorium, where his daily and constant presence is essential, and in the case of a large district like the three counties from which the Winsley Sanatorium receives its patients, the previous examination of the patient by the sanatorium medical officer is not possible.

It is now generally admitted that the use of tuberculin is a valuable aid to other therapeutic measures, and that a small proportion of patients in sanatoria who are not progressing by ordinary hygienic means should adopt this method. Of late the eslection of cases, as well as the dosage and times of injection, have been based upon the systematic determinations of the opsonic index. Patients whose indices show wide and rapid oscillations are not considered suitable for tuberculin treatment. Those who possess a persistently high-level index are in a condition of opsonic power which cannot be raised, and the risk of inducing a negative phase should not be incurred. It is the patient with the constant low index, therefore, whose chances of improvement are the greatest. It is, however, even yet premature to give definite statements as to the precise value of tuberculin in the treatment of pulmonary forms of tuberculosis.

Many other questions are surveyed in this report, such as the relation between human and bovine tuberculosis, the feeding experiments with tubercle bacilli, and the machinery for the control of tuberculous milk and meat. The English Committee has been fortunate in having entrusted their report to the authors whose names and whose work are beyond criticism and laudation.

**Resolutions adopted at Washington.**

FROM the full reports of the mighty gathering at Washington which have been given in the journals, we may assume that, successful as it was in the opening days, the éclat with which it concluded was due to the preseuce of the President, Theodore Roosevelt, who felt that no gathering could take place fraught with greater hope for the welfare of the people at large than this one. The following resolutions were passed by the Congress :—

RESOLVED—

That the attention of States and control Governments be called to the importance of proper laws for the obligatory notification by medical attendants to the proper health authorities of all cases of tuberculosis coming to their notice, and for the registration of such cases, in order to enable the health authorities to put in operation adequate measures for the prevention of the disease.

That the utmost efforts should be continued in the struggle against tuberculosis, to prevent the conveyance from man to man of tuberculous infection as the most important source of the disease.

That preventive measures be continued against bovine tuberculosis, and that the possibility of the propagation of this to man be recognised.

That we urge upon the public and upon all Governments the establishment of hospitals for the treatment of advanced cases of tuberculosis, the establishment of sanatoria for curable cases, and the establishment of dispensaries and day and night camps for ambulant cases of tuberculosis which cannot enter hospitals and sanatoria.

That this Congress endorses such well-considered legislation for the regulation of factories and workshops, the abolition of premature and injurious labour of women and children, and the securing of sanitary dwellings as will increase the resisting power of the community to tuberculosis and other diseases.

That instruction in personal and school hygiene should be given in all schools for the professional training of teachers.

That whenever possible such instruction in elementary hygiene should be entrusted to properly qualified medical instructors.

That colleges and universities should be urged to establish courses in hygiene and sanitation, and also to include these subjects among their entrance requirements, in order to stimulate useful elementary instruction in the lower schools.

That this Congress endorses and recommends the establishment of playgrounds as an important means of preventing tuberculosis through their influence upon health and resistance to disease.

**Human and Bovine Tuberculosis.**

THIS matter was discussed at a joint session of the sections on bacteriology and pathology at Washington. Dr Robert Koch reviewed the position he had adopted at the British Congress in 1901, and stated that he had never held that the human and bovine tubercle bacilli were two distinct species, but only stated that they differed from one another in certain characteristics, which were of the greatest importance in combating tuberculosis. All competent investigators now agreed that the tubercle bacilli of human origin and those of cattle differed. Dr. Koch pointed out these differences, and said that to his knowledge the bacilli of the human type had never been demonstrated in cattle, but the bacilli of the bovine type could occur in man. With few exceptions, however, these bacilli were but slightly virulent for man and remained localised. In no case of pulmonary tuberculosis had the tubercle bacillus of the bovine type been demonstrated. If further investigation should confirm this position, it is clear that we must direct our regulations for combating tuberculosis primarily against the tubercle bacilli of the human type. And yet, on the other hand, it has been shown by Dr. Nathan Raw that in the treatment of human infections, such as pulmonary tuberculosis, a tuberculin prepared from bovine sources is much more efficient than Koch's Tuberculin R, or the new tuberculin.

**Bristol General Hospital.**

A RECENT alteration of rules, which regulates the constitution of the staff of this hospital, is worthy of passing notice. The addition of the word "Skiagraphist" has been made, and accordingly the holder of the office now becomes a member of the "Honorary Staff." We congratulate Mr. John Ellington Jones on the fact that his work is being so fully appreciated, and that he himself now takes that position which he and his office so fully deserve.

